# Utility of the modified 5-item frailty index as a predictor of postoperative febrile urinary tract infection in patients who underwent ureteroscopy with laser lithotripsy

**DOI:** 10.1007/s00345-024-05016-y

**Published:** 2024-05-15

**Authors:** Shinsaku Tasaka, Yuki Kohada, Mikio Ikeda, Ryuhei Kanaoka, Mutsuo Hayashi, Nobuyuki Hinata

**Affiliations:** 1 Department of Urology, Takanobashi Central Hospital, Hiroshima, Japan; 2 Department of Urology, Hiroshima University Graduate School of Biomedical Sciences, 1-2-3 Kasumi, Minamiku, Hiroshima, 734-8551 Japan

**Keywords:** Ureteroscopy, Laser lithotripsy, Frailty, Stone free, Postoperative urinary tract infection

## Abstract

**Purpose:**

This study aimed to assess the effect of the modified 5-item frailty index on perioperative complications and surgical outcomes in patients who underwent ureteroscopy with laser lithotripsy for upper urinary tract stones.

**Methods:**

Patients who underwent ureteroscopy with laser lithotripsy for upper urinary tract stones between 2019 and 2022 were reviewed retrospectively. Assessment was performed using the modified 5-item frailty index based on medical history (hypertension, diabetes, heart failure, chronic obstructive pulmonary disease) and functional status. Patients were categorized into the high (≥ 2) and low (≤ 1) modified 5-item frailty index groups based on the frailty score. We compared the perioperative complications and surgical outcomes between the two groups.

**Results:**

Seventy-one (15.8%) and 393 (84.1%) of the 467 patients were classified into the high and low modified 5-item frailty index groups, respectively. The high modified 5-item frailty index group exhibited a significant association with increased febrile urinary tract infections compared to the low modified 5-item frailty index group [≥ 37.8 °C: 15 (20.3%) vs 13 (3.3%), p < 0.001; ≥ 38 °C: 9 (12.2%) vs 7 (1.8%), p < 0.001]. Surgical outcomes, including operative time and stone-free rate, did not differ significantly between the two groups.

**Conclusion:**

The modified 5-item frailty index is valuable for predicting postoperative complications, particularly febrile urinary tract infections, after ureteroscopy with laser lithotripsy for upper urinary tract stones. This index allows for practical preoperative risk assessment in patients who underwent ureteroscopy with laser lithotripsy.

**Supplementary Information:**

The online version contains supplementary material available at 10.1007/s00345-024-05016-y.

## Introduction

Upper urinary tract stone is a common disorder that may affect all age groups, with an incidence of 1/1000 individuals [1]. The incidence of urinary stone disease has risen worldwide, and recent studies indicate a lifetime incidence of 13% for men and 7% for women [1]. Several investigations have reported that the prevalence increases with advancing age [2, 3]. Therefore, when treating upper urinary stones, it is imperative to address frailty, a prominent characteristic of the elderly population. Briefly, frailty is defined as the loss of physiological function and reserves beyond normal aging, resulting in a state of susceptibility and diminished resistance to systemic or external stressors, such as surgical procedures [4].

Several frailty indices have been developed to facilitate the assessment of perioperative risk factors for postoperative adverse events and mortality. One of the earliest and most extensive risk assessment tools was the 70-item frailty index based on the Canadian Study of Health and Aging, which possesses high predictive value for adverse surgical outcomes [5]. Subsequently, a similar 11-items frailty index was devised based on the American College of Surgeons National Surgical Quality Improvement Program database, which was further abridged to 5 factors in the modified 5-item frailty index (mFI-5) [6]. This simplified version, which has yielded consistent predictions of postoperative complications and mortality, has been validated in different cohorts of patients undergoing various surgical procedures [7–9]. Despite the well-documented role of the mFI-5 as an accurate risk stratification tool, its applicability and utility for predicting outcomes in patients undergoing ureteroscopy with laser lithotripsy (URS) remains to be determined.

This study aimed to evaluate the influence of the mFI-5 on perioperative complications and surgical outcomes in patients who underwent URS for upper urinary tract stones.

## Participants and methods

### Database and study population

This retrospective, single-center study included patients who underwent URS at Takanobashi Central Hospital between April 2019 and March 2022. The data of all patients were anonymized with consent for inclusion in the study, which was approved by the Ethics Committee of Takanobashi Central Hospital (2023–0001). The exclusion criteria were loss to follow-up, missing data, and inaccessible urinary stone due to a narrow ureter. All patients included in this study received evidence-based perioperative antibiotics following the recommendations of the European Association of Urology guidelines, and those who did not were excluded [10].

### Surgical method

All procedures were performed in the dorsal lithotomy position under general anesthesia. Rigid URS was performed using a 4.5-Fr semi-rigid ureteroscope for accessible ureteral stones. Flexible URS (f-URS) was performed using a 7.5-Fr flexible ureteroscope for renal pelvis stones or ureteral stones that were inaccessible by semi-rigid ureteroscopy. A ureteral access sheath was placed in all patients who underwent f-URS (size 10/12 Fr). All URS procedures were performed using a holmium-yttrium aluminum garnet laser with an energy setting of 0.5 to 1.0 J and 8 to 10 Hz frequency if possible. Fragments smaller than 2 mm were left to pass, and larger ones were retrieved. The ureteral stent was removed 2–4 weeks after URS.

### Modified 5-item frailty index

The mFI-5 was used to assess frailty in the study population. This instrument is a simplified version of the 11-item frailty index that can predict postoperative complications in various surgical subspecialties [6]. Patient frailty was quantified using five items: history of four comorbidities [hypertension, diabetes, congestive heart failure (CHF), and chronic obstructive pulmonary disease (COPD)] and the level of independence when performing the activities of daily living [11]. Based on previous studies, preoperative mFI-5 scores ≥ 2 and ≤ 1 were designated as the high and low mFI-5 groups, respectively [12, 13].

### Clinical data collection

Clinical data were collected retrospectively from the patients’ medical records. The clinical variables included age, sex, body mass index, presence of hydronephrosis, stone location, stone size, number of stones, computed tomography (CT) values, preoperative urine culture, pre-stenting, and preoperative treatment of calculous pyelonephritis.

### Outcomes

The primary outcomes of this study included perioperative complications, viz. febrile urinary tract infection (f-UTI), septic shock, and ureteral stricture. The secondary outcomes were surgical outcomes, viz. operative time and the stone-free rate.

f-UTI was defined using two cut-offs of ≥ 37.8 °C and ≥ 38.0 °C, respectively. Patients who were administered antibiotics for another source of fever other than the urinary tract were excluded [14]. Septic shock was defined as f-UTI that required vasopressor agents to maintain a mean arterial pressure of 65 mmHg or higher despite adequate volume resuscitation [15]. Ureteral stricture was defined as worsening of postoperative hydronephrosis compared to baseline. Postoperative stone-free status was defined as the presence of stone fragments of 4 mm or less visualized using abdominal ultrasonography and X-ray for patients with non-lucent stones and abdominal CT for those with lucent stones.

### Statistical analysis

The distribution of categorical variables was compared using Pearson’s Chi squared test. Fisher's exact test was used if the number of events was insufficient to perform Pearson’s chi-squared test. Differences in variables with continuous distribution across dichotomous categories were assessed using Student's t test. Univariate and multivariate logistic regression analyses were performed to evaluate the predictive factors of f-UTI after URS. All data were analyzed using R 4.3.1 (R foundation for statistical computing, Vienna, Austria). Statistical significance was set at p < 0.05.

## Results

### Patient characteristics

Of the 511 eligible patients, 467 patients were included in this study. The distribution of mFI-5 scores was as follows: score 0, n = 199 (42.6%); score 1, n = 194 (41.5%); score 2, n = 56 (12.0%); score 3, n = 17 (3.6%); and score 4, n = 1 (0.2%). Of the clinical components of the mFI-5, 218 patients (46.7%) had chronic hypertension, 71 patients (15.2%) had diabetes, 26 patients (5.6%) had a history of COPD, 22 patients (4.7%) had a history of CHF, and 27 patients (5.8%) were partially or totally dependent while performing the activities of daily living (Table 1).

**Table 1:** Variables included in the modified 5-item frailty index

mFI-5 subgroups (*n* = 467)	No (%)
mFI-5:0	199 (42.6)
mFI-5:1	194 (41.5)
mFI-5:2	56 (12.0)
mFI-5:3	17 (3.6)
mFI-5:4	1 (0.2)
mFI-5:5	0 (0)
mFI-5 clinical components (*n* = 467)	No (%)
Chronic hypertension	218 (46.7)
Diabetes	71 (15.2)
Chronic obstructive pulmonary disease	26 (5.6)
Congestive heart failure	22 (4.7)
Dependent functional status	27 (5.8)

*mFI-5* modified 5-item frailty index

Based on the mFI-5 scores, 74 patients (15.8%) were allocated to the high mFI-5 group, while 393 patients (84.1%) were allocated to the low mFI-5 group. Table 2 presents the comparison of patients’ characteristics between the high and low mFI-5 groups. A high mFI-5 score was correlated with older age (p < 0.001), female predominance (p = 0.015), lower CT values (p = 0.003), urine culture positivity (p = 0.001), pre-stenting (p = 0.021), and calculous pyelonephritis (p = 0.004).

**Table 2:** Comparison of patient background between the high and low mFI-5 groups

	All (*N* = 467)	Low mFI-5 (*n* = 393)	High mFI-5 (*n* = 74)	*p* value
Age(years), average (SD)	60.7 ± 14.06	58.7 ± 13.6	71.7 ± 11.1	< 0.001*
Sex (Female, %)	172 (36.8)	135 (34.4)	37 (50.0)	0.015*
BMI (kg/m^2^), average (SD)	24.5 ± 4.4	24.5 ± 4.2	24.7 ± 5.3	0.743
Hydronephrosis (%)	290 (62.0)	243 (61.8)	47(63.5)	0.886
right/left	216/251	180/213	36/38	0.746
Location of urinary stone				0.835
Renal pelvic stone (%)	137 (29.3)	115 (29.3)	22 (29.7)	
Ureteropelvic junction stone (%)	36 (7.7)	29 (7.4)	7 (9.5)	
Upper ureteral stone (%)	173 (37.0)	143 (36.4)	30 (40.5)	
Middle ureteral stone (%)	50 (10.7)	43 (10.9)	7 (9.5)	
Lower ureteral stone (%)	71 (15.2)	63 (16.0)	8 (10.8)	
Maximum diameter (cm), average (SD)	11.9 ± 5.7	11.9 ± 5.7	11.9 ± 6.0	0.956
Multiple stones (%)	174 (37.3)	148 (37.7)	26 (35.1)	0.382
CT value (HU), average (SD)	1235 ± 354	1257 ± 344	1123 ± 389	0.003*
Urine culture (positive)	107 (22.9)	79 (20.1)	28 (37.8)	0.001*
Pre-stenting (%)	85 (18.2)	64 (16.3)	21 (28.4)	0.021*
Calculous pyelonephritis (%)	38 (8.1)	25 (6.4)	13 (17.6)	0.003*

*BMI* body mass index, *CT* computed tomography, *HU* Hounsfield unit, *mFI-5* modified 5-item frailty index

^*^p < 0.05

### Predictive ability of mFI-5 for postoperative f-UTIs

Table 3 presents the patients’ perioperative complications. The high mFI-5 group exhibited significantly higher rates of f-UTIs and septic shock. No patients died of septicemia postoperatively. Univariate and multivariate analyses were conducted to identify significant associations between postoperative f-UTIs and the mFI-5 score (Table 4). Univariate analysis showed that a high mFI-5 score, urine culture positivity, and age > 65 years were significantly associated with ≥ 37.8 °C and ≥ 38.0 °C f-UTIs. Multivariate regression analysis for ≥ 37.8 °C f-UTI showed that high mFI-5 scores [odds ratio (OR) 5.5, CI 2.1–14.2; p < 0.001] and urine culture positivity were independent predictive factors for f-UTI. These findings demonstrate the substantial predictive value of mFI-5 for f-UTI in patients who underwent URS.

**Table 3:** Comparison of postoperative complications between the high and low mFI-5 groups

	All (*N* = 467)	Low mFI-5 (*n* = 393)	High mFI-5 (*n* = 74)	*p* value
Postoperative complication (%)	39 (8.4)	23 (5.9)	16 (21.6)	< 0.001*
f-UTI over 37.8 °C (%)	28 (6.0)	13 (3.3)	15 (20.3)	< 0.001*
f-UTI over 38.0 °C (%)	16 (3.4)	7 (1.8)	9 (12.2)	< 0.001*
Septic shock (%)	3 (0.6)	0 (0)	3 (4.1)	0.004*
Ureteral stricture (%)	11 (2.4)	10 (2.5)	1 (1.4)	0.839

f-UTI: febrile urinary tract infection, mFI-5: modified 5-item frailty index

^*^p < 0.05

**Table 4:** Univariate and multivariate analyses for the risk factor of postoperative f-UTI

	Univariate analysis		Multivariate analysis	
	OR (95%CI)	*p* value	OR (95%CI)	*p* value
Pyrexia over 37.8 °C (*n* = 28)				
High mFI-5	7.4 (3.4–16.4)	< 0.001*	5.5 (2.1–14.2)	< 0.001*
Positive urine culture	8.42 (3.7–19.2)	< 0.001*	6.8 (2.9–16.1)	< 0.001*
Age > 65 years	2.47 (1.1–5.5)	0.026*	1.12 (0.4–2.9)	0.824
Over 38.0 °C (*n* = 16)				
High mFI-5	7.6 (2.8–21.2)	< 0.001*		
Positive urine culture	11.2 (3.6–35.6)	< 0.001*		
Age > 65 years	4.08 (1.3–12.9)	0.162*		

*f-UTI* febrile urinary tract infection, *OR* odds ratio, *CI* confidence interval

^*^p < 0.05

### Comparison of the surgical outcome between the high and low mFI-5 groups

Supplementary Table 1 shows the comparison of the surgical outcome between the two groups. No significant differences were observed in the operative time, stone-free rate, and additional treatment between the high and low mFI-5 groups.

## Discussion

We investigated frailty using the mFI-5 in patients who underwent URS. To the best of our knowledge, this study is the first to evaluate the influence of frailty in patients who underwent URS. A previous study reported that elderly patients who were fit for surgery did not have a higher complication rate and tended to live for long periods after surgery and recommended that they should undergo active stone treatment [16]. Therefore, assessment of conditions that are contraindications for surgery, including frailty, is necessary to decide which patients are fit to undergo URS. The prevalence of frailty among elderly adults is estimated to be 10%, and the rate of high mFI-5 in our cohort (15.8%) is comparable to the prevalence of frailty in previous studies [16–18]; thus, it can be inferred that the mFI-5 is a valid frailty assessment tool for patients who underwent URS.

The mFI-5 is classified as an accumulated deficits index rooted in the theory of accumulated deficits frailty [19, 20]. This theory is based on the conceptual framework that a global system loses robustness as it develops various illnesses or functional declines, termed “deficits,” and asserts that a system fails completely at a certain deficit threshold (e.g., death) [21]. Although physical measurements are unnecessary, the accumulated deficit index is calculated using a substantial collection of comprehensive medical and functional information. In contrast, the mFI-5 requires minimal information and minimizes the drawbacks of the accumulated deficit tool while maximizing its advantages, rendering it a valuable and convenient tool for assessing frailty in routine clinical practice.

We demonstrated a relationship between the mFI-5 and postoperative f-UTI in patients who underwent URS. Postoperative f-UTI for URS reportedly occurs in approximately 1–10% of patients, with sepsis occurring in approximately 0.3–1% [22]. In our study, the rates of f-UTIs (≥ 37.8 °C: 20.3%, ≥ 38 °C: 12.2%) and septic shock (4.1%) were higher in the high mFI-5 group than previously reported, while the low mFI-5 group exhibited rates similar to those reported in previous studies. Since no increase in postoperative f-UTI has been reported in elderly patients who underwent URS, frailty may influence the development of f-UTI rather than age [23, 24]. An association among frailty assessed by mFI-5, perioperative infections, and f-UTIs has been reported in other diseases [25–28]. These reports are consistent with our results that high mFI-5 scores are associated with postoperative f-UTI in patients who underwent URS. Since frailty also entails dysfunction of the immune system [29, 30], it may be associated with a higher rate of f-UTIs in patients with high m-FI scores. Future studies should evaluate the molecular and cellular mechanisms underlying the differential immune system between patients with and without frailty.

We demonstrated that the operative time and stone-free rate were not inferior in the high mFI-5 group compared to the low mFI-5 group, indicating that even patients with frailty who have high mFI-5 scores could benefit immensely from URS if complications can be prevented. Appropriate perioperative management, including prophylaxis against infection, and preoperative intervention for frailty, can ensure safe URS for patients with frailty.

This study has some limitations. This study incorporated a retrospective single-center design and included a relatively small sample population. The limited sample size may have led to selection bias. The small number of patients with f-UTI forced us to perform the analysis using two cut-offs for body temperature. The two cut-offs for f-UTI may be a cause of statistical bias. Moreover, because multiple surgeons performed URS in this study, there was considerable heterogeneity in the level of surgical skill and the choice of treatment by each surgeon. Additionally, different sensitivities in methods when evaluating postoperative stone-free status might cause diagnostic bias. Despite these limitations, relatively few studies have evaluated the effect of frailty assessment on the prediction of postoperative outcomes in patients undergoing upper urinary tract stone surgery. Therefore, we believe that the results of our study will be valuable in the preoperative assessment for URS in clinical practice.

## Conclusions

High mFI-5 scores emerged as a significant predictive indicator of postoperative f-UTIs in patients who underwent URS. The findings of this study may guide surgeons seeking to optimize the patient’s condition preoperatively to reduce adverse outcomes during the postoperative course.

## Supplementary Information

Below is the link to the electronic supplementary material.

Supplementary file1 (DOCX 17 KB)

## Data Availability

Data supporting the findings of this study are available from the corresponding author (YK) upon reasonable request.
