# Supplementary material for: Utility of the modified 5-item frailty index as a predictor of postoperative febrile urinary tract infection in patients who underwent ureteroscopy with laser lithotripsy
Source: World J Urol. 2024 May 15;42(1):323. doi: 10.1007/s00345-024-05016-y (PMC11096205; doi:10.1007/s00345-024-05016-y)
Supplement: Supplementary file 1 — Supplementary file1 (DOCX 17 KB) [file 345_2024_5016_MOESM1_ESM.docx]

Supplementary Table 1

|  | All (N=467) | Low mFI-5 (n=393) | High mFI-5 (n=74) | p-ratio |
| --- | --- | --- | --- | --- |
| Operative time>60 min (%) | 138 (29.6%) | 110 (28.0%) | 28 (37.8%) | 0.118 |
| Operative time, min | 51.4±21.9 | 50.7±21.3 | 54.9±24.6 | 0.130 |
| Stone-free rate (%) | 390 (83.5%) | 332 (84.5%) | 58 (78.4%) | 0.260 |
| Additional treatment |  |  |  |  |
| ESWL (%) | 19 (4.1%) | 17 (4.3%) | 2 (2.7%) | 0.743 |
| URS/RIRS (%) | 8 (1.7%) | 8 (2.0%) | 0 (0%) | 0.453 |

Surgical outcomes

URS: ureteroscopy, RIRS: retrograde intrarenal surgery, ESWL: extracorporeal shockwave lithotripsy, mFI-5: modified 5-item frailty index

*p < 0.05
